# Exploiting Mannuronan C-5 Epimerases in Commercial Alginate Production

**DOI:** 10.3390/md18110565

**Published:** 2020-11-18

**Authors:** Anne Tøndervik, Olav A. Aarstad, Randi Aune, Susan Maleki, Philip D. Rye, Arne Dessen, Gudmund Skjåk-Bræk, Håvard Sletta

**Affiliations:** 1 Department of Biotechnology and Nanomedicine, SINTEF Industry, Richard Birkelands vei 3B, N-7034 Trondheim, Norway; Randi.Aune.bio@sintef.no (R.A.); susan.maleki@sintef.no (S.M.); Havard.Sletta@sintef.no (H.S.); 2 Department of Biotechnology and Food Science, Norwegian University of Science and Technology, NTNU, Sem Sælands vei 6-8, N-7491 Trondheim, Norway; olav.a.aarstad@ntnu.no (O.A.A.); gudmund.skjak-brak@ntnu.no (G.S.-B.); 3 AlgiPharma AS, Industriveien 33, N-1337 Sandvika, Norway; phil.rye@algipharma.com (P.D.R.); arne.dessen@algipharma.com (A.D.)

**Keywords:** seaweed and bacterial alginate, mannuronan C-5 epimerases, guluronate oligomers, potentiation of antibiotic effect

## Abstract

Alginates are one of the major polysaccharide constituents of marine brown algae in commercial manufacturing. However, the content and composition of alginates differ according to the distinct parts of these macroalgae and have a direct impact on the concentration of guluronate and subsequent commercial value of the final product. The *Azotobacter vinelandii* mannuronan C-5 epimerases AlgE1 and AlgE4 were used to determine their potential value in tailoring the production of high guluronate low-molecular-weight alginates from two sources of high mannuronic acid alginates, the naturally occurring harvested brown algae (*Ascophyllum nodosum, Durvillea potatorum*, *Laminaria hyperborea* and *Lessonia nigrescens*) and a pure mannuronic acid alginate derived from fermented production of the mutant strain of *Pseudomonas fluorescens* NCIMB 10,525. The mannuronan C-5 epimerases used in this study increased the content of guluronate from 32% up to 81% in both the harvested seaweed and bacterial fermented alginate sources. The guluronate-rich alginate oligomers subsequently derived from these two different sources showed structural identity as determined by proton nuclear magnetic resonance (^1^H NMR), high-performance anion-exchange chromatography with pulsed amperometric detection (HPAEC-PAD) and size-exclusion chromatography with online multi-angle static laser light scattering (SEC-MALS). Functional identity was determined by minimum inhibitory concentration (MIC) assays with selected bacteria and antibiotics using the previously documented low-molecular-weight guluronate enriched alginate OligoG CF-5/20 as a comparator. The alginates produced using either source showed similar antibiotic potentiation effects to the drug candidate OligoG CF-5/20 currently in development as a mucolytic and anti-biofilm agent. These findings clearly illustrate the value of using epimerases to provide an alternative production route for novel low-molecular-weight alginates.

## 1. Introduction

Alginates are anionic and linear polysaccharides composed of 1–4 linked residues of β-D-mannuronic acid (M) and its C-5 epimer α-L-guluronic acid (G) [1,2]. Alginates have no regular repeating structure throughout the entire polymer chains; however, the monomers can be arranged in blocks of G, M and MG of varying length and distribution [3,4]. The overall content and distribution of the monomers in these block structures determine the physicochemical and rheological properties of the alginates [5]. Alginates show a strong affinity for divalent cations like Ca^2+^, a property largely due to selective binding to G-blocks, while MG- and M-blocks are also able to bind Ca^2+,^ but to a much less degree [6]. Strong hydrogels formed by Ca^2+^-mediated intra- and intermolecular crosslinking of alginate molecules containing G-blocks represents one of the most frequent traditional uses of alginates in a variety of different food, cosmetic and biomedical applications [7,8]. Alginates are also used in medical and pharmaceutical applications like anti-reflux drugs and dressings for wound healing, where the gelling and water-binding properties are of vital importance [9]. Consequently, alginates containing G-blocks are regarded as the most relevant structures.

Alginates are one of the major polysaccharide constituents of brown algae like *Laminaria hyperborea* and *Ascophyllum nodosum*, the former being the main source for current commercial manufacturing of alginate. The content and composition of alginate differ according to the distinct parts of the algae. For example, the stipe and leaves of *L. hyperborea* contain alginate with about 68% G and 55% G, respectively [3]. Other brown algal species (e.g., *L. digitata*, *A. nodosum*, *Durvillea potatorum*, *Lessonia nigrescens*, *Saccharina latissima* and *Alaria esculenta*) contain alginate with a lower level of G content up to 50% [7]. Alginate is also produced by two bacterial genera: *Pseudomonas* sp. produces alginate containing single G residues (i.e., no G-blocks) presumably as a pathogenicity factor involved in biofilm formation, whereas *Azotobacter* sp. produces alginate containing a variety of structures including G-blocks that provide an important part of cyst formation in the complex life-cycle of the bacteria [10]. In both brown algae and bacteria, alginates are first synthesized as a polymer containing only mannuronan, usually denoted as M residues. Certain M residues are then converted to G by mannuronan C-5 epimerases, creating different M/G patterns dependent on the activity of the specific enzyme. Most of the knowledge concerning alginate biosynthesis is at present available from the soil bacterium *Azotobacter vinelandii*, *Pseudomonas fluorescens* and *Pseudomonas aeruginosa,* the latter being a serious human pathogen for people with cystic fibrosis (CF). *A. vinelandii* encodes a family of seven secreted mannuronan C-5 epimerases (AlgE1-AlgE7) which are structurally homologous but give rise to different alginate structures. AlgE4 produces strictly alternating MG sequences, whereas AlgE1 and AlgE6 are G-block forming enzymes, with AlgE1 creating the longest G-blocks [11,12]. AlgE1-AlgE7 have been subjected to extensive studies in terms of activity, structural analysis and mode of action [13]. Due to the generation of various epimerization patterns, these bacterial mannuronan C-5 epimerases have been highlighted as important tools for tailoring alginate structures desired for specific applications. In recent years, a few epimerases from brown algae have been subject to heterologous recombinant expression, but a thorough characterization of the epimerization patterns generated by these enzymes is still not available [14,15]. The principle of tailoring using the bacterial epimerases has been demonstrated with alginates for cell encapsulation where microbeads prepared by in vitro epimerization displayed superior properties compared to native algal alginates when it comes to elasticity, permeability and stability [16]. This tailoring approach has also been used in the preparation of RGD-grafted alginates for 2D or 3D culturing of human cell lines [17], whereby alginate was chemically modified before the introduction of G-blocks by epimerization to allow for gel formation of the grafted material. Furthermore, sulfated alginates have been shown to have heparin-like properties and the ability to perform precise chemical modifications resulted in advantageous biological properties when using an alternating MG alginate structure prepared by in vitro epimerization using AlgE4 [18].

OligoG CF-5/20 is a low-molecular-weight alginate rich in guluronate residues and is currently in phase II clinical trials for the treatment of CF lung disease as a dry powder for inhalation. OligoG is prepared from *L. hyperborea* stipe alginate by hydrolysis and fractionation, resulting in a mixture of oligomers enriched in G residues (>85%) with an average degree of polymerization between 15 and 17 (DPn). OligoG has been demonstrated to interact with mucins having an impact on mucus rheology [19,20,21,22,23], and recent studies suggest that OligoG can mobilize stagnant CF mucus by a calcium chelating mechanism [24]. Furthermore, OligoG is able to reduce and disrupt biofilm formation of the important CF lung pathogen *P. aeruginosa* presumably by direct disruption of the extracellular matrix and indirectly by modulating quorum sensing pathways [25,26,27]. OligoG also enhances the effectiveness of antibiotics for a number of Gram-negative (including *P. aeruginosa*) and Gram-positive bacterial and fungal pathogens [28,29,30].

As mentioned above, current manufacturing of OligoG is based on *L. hyperborea* stipe alginate obtained from raw material harvested mainly in the north Atlantic Ocean. The OligoG in development for treating CF is currently manufactured from alginate extracted from harvested seaweed, with further processing to obtain highly purified oligosaccharides. This seaweed source is acceptable for a small volume niche market, such as CF. However, the volume demanded for larger disease indications such as chronic obstructive pulmonary disease (COPD), or wound care would dramatically outstrip the supply currently available from harvested seaweed sources. Clearly, there is a need to identify commercially viable alternative sources.

In this paper, we present a strategy for the preparation of OligoG and, in principle, also polymeric material for other applications requiring alginates with a high level of guluronate by utilizing bacterially produced alginate and mannuronan C-5 epimerases with specific activities for tailoring the final product. Furthermore, we demonstrate that seaweed alginates with an intrinsic low level of guluronate can be “upgraded” by enzymatic treatment and used for OligoG production. This highlights the possibility of increased utilization of the *L. hyperborea* biomass already harvested as well as other algal resources.

## 2. Results and Discussion

### 2.1. Seaweed Alginates Rich in Mannuronic Acid Can Be Upgraded and Used for Production of Guluronate Oligomers

Alginate extracted from the stem of *Laminaria hyperborea*, which is the present source of OligoG CF-5/20, generally contains 60–70% guluronic acid residues, whereas the leaf alginate from the same species has around 50% guluronic acid. Alginates extracted from other brown algal species such as *Lessonia nigrescens*, *Ascophyllum nodosum* and *Durvillea potatorum* have lower levels of guluronic acid (up to 50%) and are thus not suited for making G-rich oligomers. However, it should be possible to increase the G-content of these alginates considerably by in vitro epimerization making them potential sources for OligoG production. To explore this, alginates from the above-mentioned seaweed species were epimerized with the G-block forming enzymes AlgE1 and AlgE6 alone and in combination with AlgE4. The G-content and level of dyads (GG) and triads (GGG) in the alginates before and after epimerization were determined by NMR (complete results in Supplementary Table S1).

**Figure 1:**
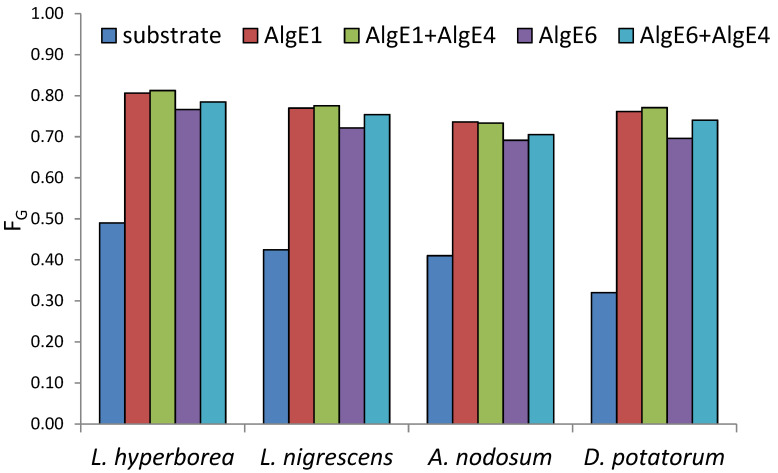
Content of guluronic acid (F_G_ determined by NMR analyses) in seaweed alginate from different species before (blue) and after epimerization with AlgE1 and AlgE6 alone (red and purple bars) and in combination with AlgE4 (green and turquoise bars).

All alginates were epimerized to high levels of G after treatment with mannuronan C-5 epimerases (Figure 1). *L. hyperborea* (leaf), which is the material with the highest G, reached 81% G after epimerization both with AlgE1 alone and in combination with AlgE4. The use of AlgE1 either alone or together with AlgE4 resulted in the highest level of G for all substrates. This is likely due to AlgE1 being a bifunctional enzyme efficient at elongating the G-blocks that are already present in the algal alginate substrates as well as condensing G-blocks by epimerizing M residues flanked by two G residues [12].

**Figure 2:**
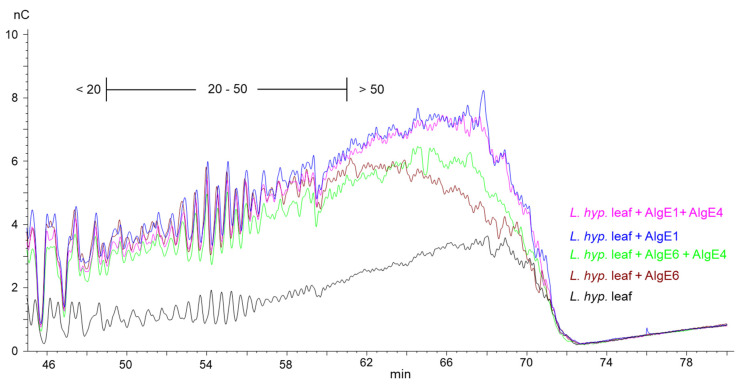
Section of HPAEC-PAD chromatograms of alginate samples from *L. hyperborea* leaf before (black line) and after epimerization with AlgE1 (blue line) and AlgE6 (brown line) alone and in combination with AlgE4 (pink and green lines). The epimerized alginates were degraded with M-lyase prior to analysis, thus leaving only the G-blocks. The scale bar above the chromatograms indicates the degree of polymerization (DP) eluting at different time points, i.e., DP below 20, DP 20–50 and DP > 50.

This is shown in the HPAEC-PAD chromatograms of epimerized alginate from *L. hyperborea* leaf that were degraded with M-lyase (alginate lyase from *Haliotis tuberculate* cleaving M-M and M-G linkages) after epimerization, thus leaving only the G-blocks. AlgE1 and AlgE1 + AlgE4 gave slightly longer G-blocks than AlgE6 and AlgE6 + AlgE4 as visualized in the curves extending most far to the right in the chromatogram in Figure 2 (i.e., G-blocks eluting from approximately 60 min and later). AlgE1 and AlgE1 + AlgE4 also resulted in a higher level of the longest G-blocks, which is shown by the higher response (nC) in the chromatograms. The same effect was observed for the samples from *L. nigrescens* (Supplementary Figure S1).

**Figure 3:**
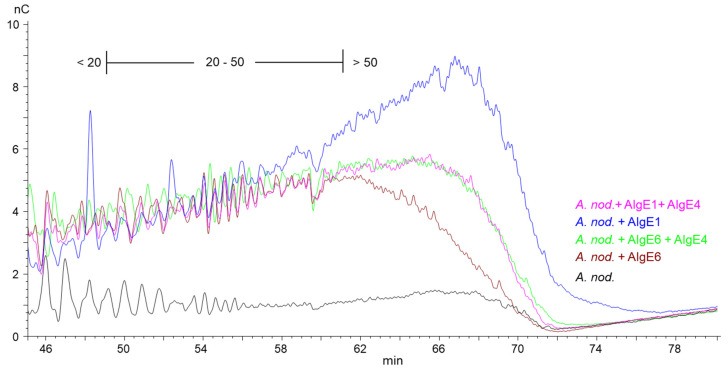
Section of high-performance anion-exchange chromatography with pulsed amperometric detection (HPAEC-PAD) chromatograms of alginate samples from *A. nodosum* before (black line) and after epimerization with AlgE1 (blue line) and AlgE6 (brown line) alone and in combination with AlgE4 (pink and green lines). The epimerized alginates were degraded with M-lyase prior to analysis, thus leaving only the G-blocks. The scale bar above the chromatograms indicates the degree of polymerization (DP) eluting at different time points, i.e., DP below 20, DP 20–50 and DP > 50.

However, for *A. nodosum* (Figure 3) and *D. potatorum* (Supplementary Figure S2), it appears that AlgE1 combined with AlgE4 give shorter G-blocks and lower levels of long G-blocks than AlgE1 alone even though the total G-content was very similar in the two cases (Figure 1). The reason for this is not known but may be due to AlgE4 sterically blocking access to the substrate or the processive action of AlgE1 on these types of alginates.

**Figure 4:**
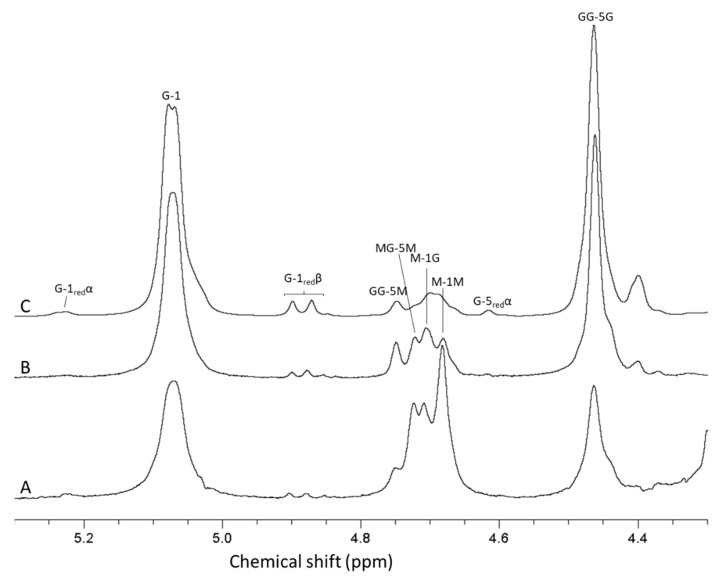
1H-NMR spectra showing alginate from *Laminaria hyperborea* leaf (**A**), after epimerization by AlgE4 and AlgE1 (**B**) and oligomers produced by hydrolysis of the epimerized alginate (**C**). The content of guluronic acid in the three materials are 0 (**A**), 79 (**B**) and 90% (**C**), respectively. AB-nC denotes proton n in uronic acid B with neighboring uronic acids A and C., e.g., MG-5 M is the resonance from H 5 in guluronic acid between two mannuronic acid units.

**Figure 5:**
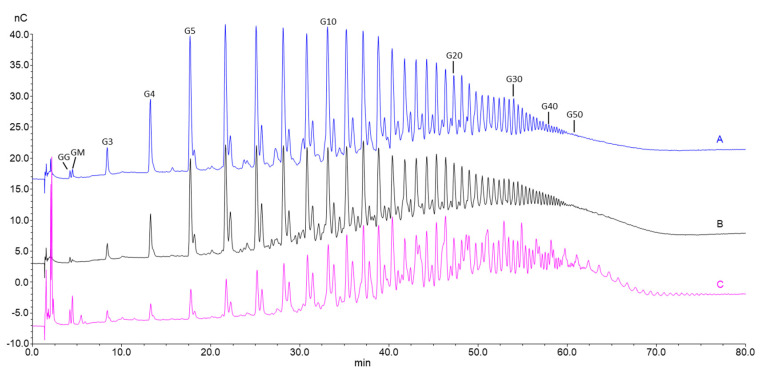
Overlayed HPEC-PAD chromatograms of oligomers produced by hydrolysis of bacterial mannuronan first epimerized with AlgE4 and AlgE1 (**A**), alginate from *L. hyperborea* leaf epimerized with AlgE4 and AlgE1 (**B**) and OligoG CF-5/20 (**C**). Numbers above the peaks indicate the oligomer length (degree of polymerization: DP).

For analysis of antibiotic potentiation properties (see below), one batch of guluronate oligomers was prepared by epimerization of alginate from *L. hyperborea* leaf using AlgE1 and AlgE4 in combination (1000 U/g and 400 U/g alginate of AlgE4 and AlgE1, respectively). The resulting alginate reached 79% G (determined by NMR) and was hydrolyzed and precipitated, yielding oligomers with 90% G and a molecular weight average molecular weight of 4.5 kDa (determined by SEC-MALS). NMR spectra of alginate before and after epimerization and the oligomers produced are shown in Figure 4. The HPEC-PAD chromatograms of these oligomers are shown in Figure 5B. It should be noted that the peaks in the OligoG CF-5/20 chromatogram eluting around 60 min were due to oligomers enriched in M residues. Furthermore, the complexity in the same chromatogram arose from G oligomers that had internal M residues and thus had slightly different elution profiles than pure G oligomers of a corresponding length.

### 2.2. Guluronate Oligomers Prepared from Bacterially Produced Mannuronan

**Figure 6:**
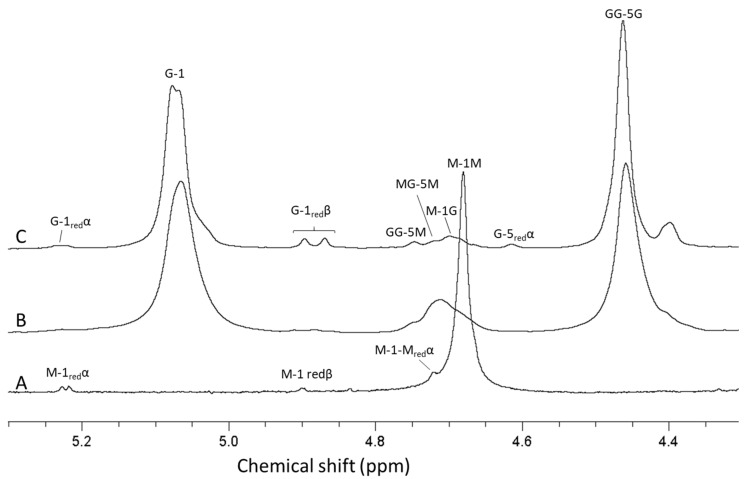
NMR spectra showing mannuronan produced by *P. fluorescens* (**A**), mannuronan epimerized by AlgE4 and AlgE1 (**B**) and oligomers produced by hydrolysis of the epimerized mannuronan (**C**). The contents of guluronic acid in the three materials are 0 (**A**), 80 (**B**) and 93% (**C**), respectively. AB-nC denotes proton n in uronic acid B with neighboring uronic acids A and C., e.g., MG-5M is the resonance from H 5 in guluronic acid between two mannuronic acid units.

Mannuronan produced by *Pseudomonas fluorescens* strain Pf20118 was isolated from the culture supernatant after fermentation by precipitation with acid and isopropanol. Acid precipitation is needed to remove an acid-soluble polymer additionally produced by *P. fluorescens*. The alginate yield in the fermentation process with the given conditions are generally around 10 g/L fermentation broth. The molecular weight of mannuronan produced by this process is in the range of 100–150 kDa, as determined by SEC-MALS. Based on previous work from the authors’ research groups, AlgE4 and AlgE1 were used sequentially for the epimerization of the bacterial mannuronan substrate. This is due to AlgE1 showing high efficiency of epimerization and introducing long G-blocks on an MG-alternating substrate [12]. Additionally, during hydrolysis, the MG-blocks in the epimerized alginate will be degraded about 10 times faster than the G-blocks and essentially removed during acid precipitation due to higher solubility at low pH, resulting in guluronate oligomers of high purity [31]. NMR analyses showed that sequential epimerization of mannuronan with AlgE4 (1000 U/g) and AlgE1 (400 U/g) resulted in an alginate containing 72% guluronic acid. A further increase was achieved by the addition of extra CaCl_2_ (2 mM) in the AlgE1 epimerization step, which yielded alginate with 80% guluronic acid. NMR analysis of the hydrolyzed oligomers showed a G-content of 93% that was an increase compared to the starting material (Figure 6).

This was a consequence of different hydrolysis rates for the four glycosidic bonds in alginate (K_GM_ >> K_MG_ ≈K_MM_ > K_GG_), causing M and MG blocks to be hydrolyzed into small oligomers faster, and were not precipitated in the recovery step after hydrolysis and therefore lost. A G-content of 93% is within the current specifications required for OligoG CF-5/20 produced from *L. hyperborea* (>85% G). The oligomer distribution, as measured by HPAEC-PAD (Figure 5A), was highly similar to the seaweed-based OligoG CF-5/20 (Figure 5C). Furthermore, the oligomers had a higher purity than OligoG CF-5/20, as can be seen from the narrower peak width since these contain some degree of internal M residues. SEC-MALS analyses showed that the molecular weight (M_w_, weight average) of the oligomers were similar to OligoG CF-5/20, i.e., 5 kDa (corresponding to a number average molecular weight, Mn, of 3.7 kDa). The level of sodium in the oligomer material was also similar to OligoG CF-5/20 (10% or 0.1 mg Na/mg oligomer), as determined by ICP-MS analyses. An undesirable consequence of using a Gram-negative bacterial production host is the high level of endotoxins. Generally, the endotoxin content of mannuronan produced by *P. fluorescens* is in the range of 2–4 × 10^6^ EU/g as measured by LAL (limulus amebocyte lysate) based on equivalent recombinant factor C assays. However, purification with activated carbon under basic conditions reduced the level of endotoxins to <10 EU/g. Likewise, the level of protein in the oligomers was reduced to <2% by this process compared to >5% in the mannuronan starting material. The yield of the entire process starting from mannuronan was approximately 50–60%, where the majority of the loss was due to the precipitation steps after epimerization and hydrolysis (data not shown). An overview of properties of the guluronate oligomers produced from in vitro epimerized mannuronan and seaweed alginate, as well as the starting materials, is shown in Table 1.

### 2.3. Guluronate Oligomers Prepared by In Vitro Epimerization Potentiate the Effect of Antibiotics Similar to OligoG CF-5/20

OligoG CF-5/20 has been shown to potentiate the effect of a range of antibiotics on a variety of bacterial and fungal strains [28,29]. To verify that the oligomers produced from *P. fluorescens* mannuronan and upgraded seaweed alginate are functionally equivalent to OligoG CF-5/20, minimum inhibitory concentration assays were performed. *P. aeruginosa* (PAO1) and *Acinetobacter baumannii*, which are important pathogens in CF, were chosen as test organisms and aztreonam, ciprofloxacin and ceftazidime commonly used to treat these infections were the selected antibiotics. As shown in Table 2, the oligomers produced by in vitro epimerization were able to potentiate antibiotics at the same level as OligoG CF-5/20. These data show that mannuronan C-5 epimerases can be used in both bacterially fermented alginates and seaweed alginates with low content of guluronic acid as an alternative source for the production of G-rich low-molecular-weight alginate oligomers.

## 3. Materials and Methods

### 3.1. Bacterial Production and Isolation of Mannuronan

For the production of mannuronan, *Pseudomonas fluorescens* Pf20118 was used. Pf20118 is a mucoid strain derived from *P. fluorescens* NCIMB 10525 harboring mutations in *mucA* and *algG,* which activates mannuronan production and inactivates the periplasmic C-5 epimerase AlgG. Pf20118 was cultivated in 3 L Applikon fermenters using a medium containing fructose (60 g/L), yeast extract (12 g/L), (NH_4_)_2_SO_4_·H_2_O (0.6 g/L), Na_2_HPO_4_·2H_2_O (2 g/L), NaCl (11.7 g/L) and MgSO_4_·7H2O (0.3 g/L) with pH adjusted to 6.8. The cultivation temperature was 25 °C. Airflow was 0.45 VVM from the start and adjusted to 0.85 VVM 10 h after inoculation. Dissolved oxygen was maintained at a constant level of 20% of saturation by automatic adjustment of stirrer speed. To avoid degradation of mannuronan by extracellular alginate lyases, proteases (Neutrase and Alcalase, NovoZymes) were added at a final concentration of 0.1 g/L. Fermentation was ended when the carbon source was depleted, formalin was added for conservation (final concentration of 1%), and cultures were stored at 4 °C until isolation of mannuronan [32]. Cultures were diluted with 0.2 M NaCl before centrifugation (10,000 × *g*, 60 min) to remove bacterial cells. The alginate in the supernatant was deacetylated by mild alkaline treatment, as described previously [33]. The alginate was then precipitated by adding HCl (3 M) to pH < 2. The alginate was collected by centrifugation (8000× *g*, 15 min) and washed with 0.05 M HCl. The precipitated alginate was dissolved in water with NaOH (3 M) to pH 6.9–7.2. NaCl was added to a final concentration of 2 g/L, and then isopropanol 60% (*v/v*) was added to precipitate mannuronan followed by centrifugation (6000× *g*, 10 min) and air drying.

### 3.2. Seaweed Alginates

The seaweed alginates used in this study were from *Ascophyllum nodosum*, *Durvillea potatorum*, *Laminaria hyperborea* and *Lessonia nigrescens* originally obtained from Protan and characterized by NMR before in vitro epimerization (pre/post epimerization properties shown in Supplementary Table S1; molecular weights shown in Supplementary Table S2).

### 3.3. Epimerization of Alginate by Mannuronan C-5 Epimerases

Bacterial or seaweed sourced mannuronan were epimerized with different combinations of mannuronan C-5 epimerases AlgE1, AlgE4 and AlgE6. The epimerases used were expressed in recombinant strains of *E. coli* using RV308 as the expression host. Enzyme production was performed by high cell density fermentation, as described previously [34] and partial purification was performed using standard column chromatography. Epimerase activity of the purified enzymes was determined as described previously [35,36], and protein concentration was measured at 280 nm using a NanoDrop UV-vis spectrophotometer (Thermo Scientific, Waltham, MA, USA). The specific activity (U/mg protein) of the enzyme preparations used were as follows: AlgE1 (132 U/mg), AlgE4 (331 U/mg) and AlgE6 (168 U/mg). Epimerization reactions were performed with 3600, 8900 and 2700 U/g alginate for AlgE1, AlgE4 and AlgE6, respectively, in a buffer containing MOPS (40 mM), NaCl (100 mM), CaCl_2_ (2.5 mM) and spectinomycin (0,2 mg/mL), pH 6.9 at 37 °C. After epimerization, calcium was removed by the addition of EDTA (4 mM), and the alginate solution was dialyzed in water to reduce conductivity (<5 µS/cm) before freeze-drying. Alternatively, the alginate was precipitated by reducing pH with 1 M HCl (pH < 2). The precipitated material was washed twice with 0.05 M HCl and dissolved in water by neutralization with NaOH (pH 7), then freeze-dried.

### 3.4. Production of Oligomers by Acid Hydrolysis of High Molecular Weight Alginate

High molecular weight alginates (epimerized mannuronan and seaweed alginate) were hydrolyzed to oligomers by treatment with HCl in two steps. The alginate was dissolved in water (2.5 g/L) and adjusted to pH 5.6 before incubation at 95 °C for 3.5 h. Solutions were cooled to room temperature and adjusted to pH 3.6 before incubation at 95 °C for a further 8 h [37]. Alginate oligomers were precipitated with HCl (pH < 2) adjusted to pH 7 with NaOH and then freeze-dried.

### 3.5. Characterization and Purification of Alginate Oligomers

The epimerized alginates and the alginate oligomers were analyzed by NMR to determine the final G-content. ^1^H NMR spectra were recorded on a BRUKER AVANCE III HD (Bruker, Billerica, MA, USA) 400 MHz at 82 °C. Signal assignment and data processing was based on previous work [38]. Alginate oligomers were analyzed without pretreatment, while high molecular weight alginate samples were subjected to a two-step mild acid hydrolysis at pH 5.6 and pH 3.8 in order to reduce viscosity [33]. Acid hydrolyzed and lyase degraded alginates were analyzed by high-performance anion-exchange chromatography with pulsed amperometric detection (HPAEC-PAD) as a qualitative method for comparison of G block distribution between samples and in some cases also to determine the oligomer size distribution. The chain length of G-oligomers were determined by comparison with a G-block standard as described previously [11]. Alginates were degraded by *Haliotis tuberculata* M-lyase, as previously described [39]. Briefly, 25 µL samples (1 mg/mL) were injected into an ICS-5000+ system (Thermo Scientific, Waltham, MA, USA) with a 4 × 50 mm IonPac AG4A guard and a 4 × 250 mm AS4A main column using isocratic 0.1 M NaOH and a linear sodium acetate gradient of 8.75 mM/min at a flow of 1 mL/min. From the HPAEC-PAD chromatograms, the weight and number average molecular weight of acid hydrolysates with F_G_ ≥ 0.9 and Mn ≤ 5 kDa could be determined using previously reported response factors [37]. Molecular weight for acid hydrolysates and non-degraded alginates were determined by size-exclusion chromatography (SEC) with online multi-angle static laser light scattering (MALS) on TSK 4000 and 2500 PWxl columns (Tosoh Bioscience, Tokyo, Japan). Detection was by Dawn HELEOS-II multi-angle laser light scattering photometer (Wyatt Technology, Santa Barbara, CA, USA) (λ0 = 663.8 nm) followed by Optilab T-rEX differential refractometer. All samples were filtered (0.22 μm) before injection. Samples (5–10 mg/mL, injection vol. 100–500 µL) were eluted with 0.15 M NaNO_3_/0.01 M EDTA, pH 6.0) with a flow of 0.5 mL/min. The data were collected and processed (with dn/dc = 0.150 mL/g) using the Astra (v. 7.1) software (Wyatt Technology, Santa Barbara, CA, USA).

The content of inorganic matter of alginate oligomers was determined by ICP-MS as follows: all standard solutions and dilutions were prepared using ultra-purified water 18.2 MΩ from a Smart2Pure system (Thermo Scientific, Waltham, MA, USA) and HNO_3_ purified by a Savillex DST-100 acid purification system (Savillex, Minneapolis/St.Paul, MN, USA). Standards for calibration curves were prepared in 5% HNO_3_ (*v/v*) from individual stock solutions containing 1000 ug/mL each of the relevant elements (Inorganic Ventures, USA). Indium (In) was used as internal standard, and elements were measured by an Agilent 8800 Triple Quadrupole ICP-MS (Agilent Technologies, Santa Clara, CA, USA) with an SPS4 autosampler (Agilent Technologies, USA) and a standard sample introduction system (Micro Mist glass concentric nebulizer, quartz double pass spray chamber, quartz torch with 2.5 mm id and standard nickel cones). He and O_2_ modes were used in this method. Tuning conditions are shown in Supplementary Table S3. Removal of bacterial endotoxins was performed as described previously [40]. Endotoxin levels before and after purification of oligomers were determined by the PyroGene Recombinant Factor C assay (Lonza). Protein content was determined by the Micro BCA protein assay kit (Thermo Scientific).

### 3.6. Minimum Inhibitory Concentration Assays

OligoG CF-5/20 and the low-molecular-weight alginate oligomers prepared in this study were dissolved in Mueller–Hinton broth (MH broth, LabM) to 1.25 times of the desired assay concentrations (3%). Antibiotics were dissolved in MH broth with and without alginate oligomers at 1.25 times the highest desired assay concentration. Pharmaceutical grade antibiotics were purchased from Sigma-Aldrich. OligoG CF-5/20 was provided by AlgiPharma AS, Sandvika, Norway. Two-fold serial dilutions of antibiotics were made in MH-broth with different concentrations of alginate oligomers and added to four parallel wells in Nunc 384-well microplates (30 µL per well in Nunc 242757 microplates). A group of 8 wells with no addition of antibiotics for each alginate oligomer concentration was included on each microplate as a growth reference. On the day of analysis, overnight TSB cultures (inoculated from frozen stock, 6 mL in 50 mL tubes rotated at a 45-degree angle, 200 rpm, 2.5 cm amplitude, at 37 °C) were diluted further in TSB to 0.1 OD_600_ then further diluted 1:40 in MH broth. The 384-well assay plates were inoculated with 7.5 µL of the diluted culture and incubated without shaking at 37 °C. The OD_600_ was measured after approximately 19 h of incubation, and the relative growth yield was calculated based on the reference groups. The MIC value was determined as the highest concentration giving less than 30% growth in all 4 parallel wells within the sample groups.

## 4. Conclusions

The guluronate oligomers produced in the present study were based on bacterially produced mannuronan and enzymes (mannuronan C-5 epimerases AlgE1 and AlgE4) or on low G seaweed alginates subject to the same enzymatic treatment. The oligomers were shown to be chemically and functionally equivalent to OligoG produced by the existing current commercial process. This work was performed to establish and optimize protocols for producing oligomers with high levels of guluronate and a given molecular weight distribution. Although yields were around 50–60% in the total process, there is potential for improvement by optimizing recovery steps after epimerization and hydrolysis. The findings from this study show that enzymatic treatment with mannuronan C-5 epimerases represents an alternative route for the production of high G alginate products and highlights bacterial mannuronan as a reproducible and defined material for tailoring of specific alginate structures.

## Figures and Tables

**Table 1:** Properties of OligoG CF-5/20, oligomers produced from epimerized mannuronan and *L. hyperborea* leaf alginate and the starting materials used. The guluronic acid content was determined by NMR, and the weight average molecular weight (Mw) and degree of polymerization were determined by size-exclusion chromatography with online multi-angle static laser light scattering (SEC-MALS) or by integration of HPEC-PAD chromatograms. n.d.: not determined.

	Guluronic Acid Content (%)	Molecular Weight (kDa)	DPn
Mannuronan	0	100–150	n.d.
alginate from *L. hyperborea* leaf	49	250	n.d.
OligoG CF-5/20	90–95	5	15–17
oligomers prepared from epimerized *L. hyperborea* leaf alginate	90	6.5	20
oligomers prepared from epimerized mannuronan	93	5.2	17

**Table 2:** Minimum inhibitory concentration (MIC) (given in µg/mL) for antibiotics alone and in combination with 3% OligoG CF-5/20, oligomers produced by epimerization and hydrolysis of bacterial mannuronan and oligomers produced from epimerized and hydrolyzed alginate from *L. hyperborea* leaf.

		*Pseudomonas aeruginosa*			*Acinetobacter baumannii*		
Antibiotic	Oligomer Conc. (%)	OligoG CF-5/20	Oligomers Prepared from Mannuronan	Oligomers Prepared from *L. hyperborea* Leaf Alginate	OligoG CF-5/20	Oligomers Prepared from Mannuronan	Oligomers Prepared from *L. hyperborea* Leaf Alginate
Aztreonam	0	4	4	4	256	256	256
	3	2	2	2	128	128	128
Ciprofloxacin	0	0.125	0.125	0.125	2	4	4
	3	0.063	0.031	0.063	<0,5	<0.5	<0.5
Ceftazidime	0	2	2	2	>512	>512	>512
	3	1	1	1	256	512	256

## Supplementary Materials

The following are available online at https://www.mdpi.com/1660-3397/18/11/565/s1, Figure S1: HPEC-PAD chromatograms of alginate samples from *L. nigrescens* before and after epimerization with AlgE1 and AlgE6 alone and in combination with AlgE4. Figure S2: HPEC-PAD chromatograms of alginate samples from *D. potatorum* before and after epimerization with AlgE1 and AlgE6 alone and in combination with AlgE4. Table S1: Composition and sequential parameters of seaweed alginates before and after epimerization with AlgE1 and AlgE6 alone and in combination with AlgE4. Table S2: Molecular weight (analyzed by SEC-MALS) of alginates from brown algae used for epimerization. Table S3: Agilent 8800 Series Triple Quadrupole ICP-MS System parameters.
