# Supplementary material for: Exploiting Mannuronan C-5 Epimerases in Commercial Alginate Production
Source: Mar Drugs. 2020 Nov 18;18(11):565. doi: 10.3390/md18110565 (PMC7698916; doi:10.3390/md18110565)
Supplement: Supplementary file 1 [file marinedrugs-18-00565-s001.pdf]

Supplementary material

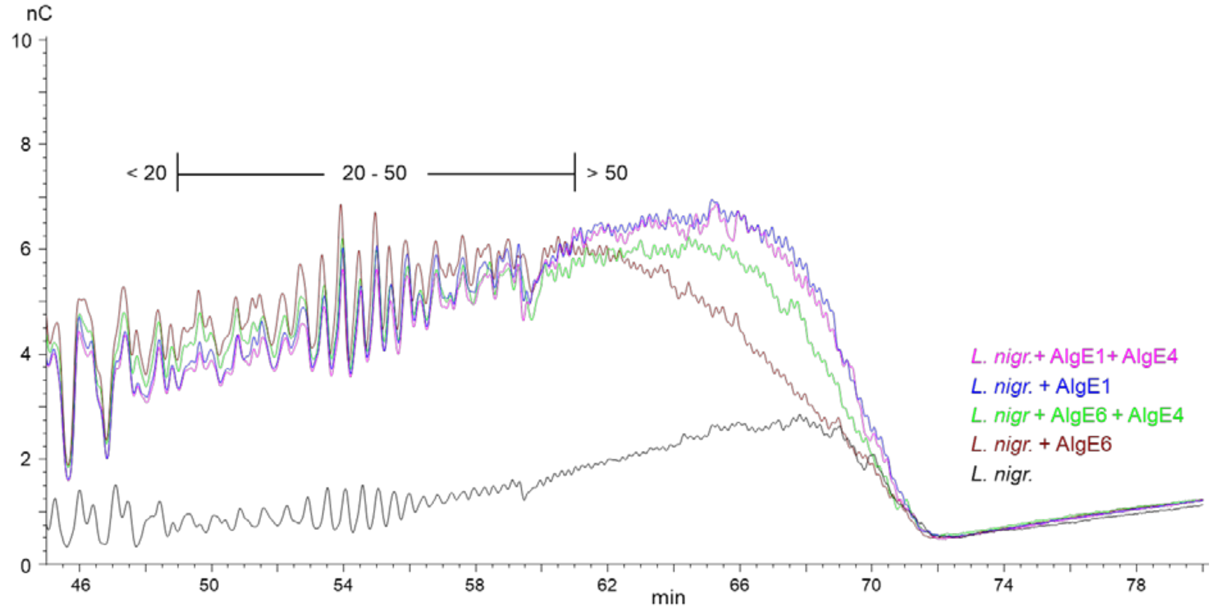

Figure S1. HPAEC-PAD chromatograms of alginate samples from *L. nigrescens* before (black line) and after epimerisation with AlgE1 (blue line) and AlgE6 (brown line) alone and in combination with AlgE4 (pink and green lines). The epimerised alginate has been degraded with M-lyase prior to analysis thus leaving only the G-blocks. The scale bar above the chromatograms indicate the degree of polymerization (DP) eluting at the different time points i.e. DP below 20, DP 20-50 and DP > 50.

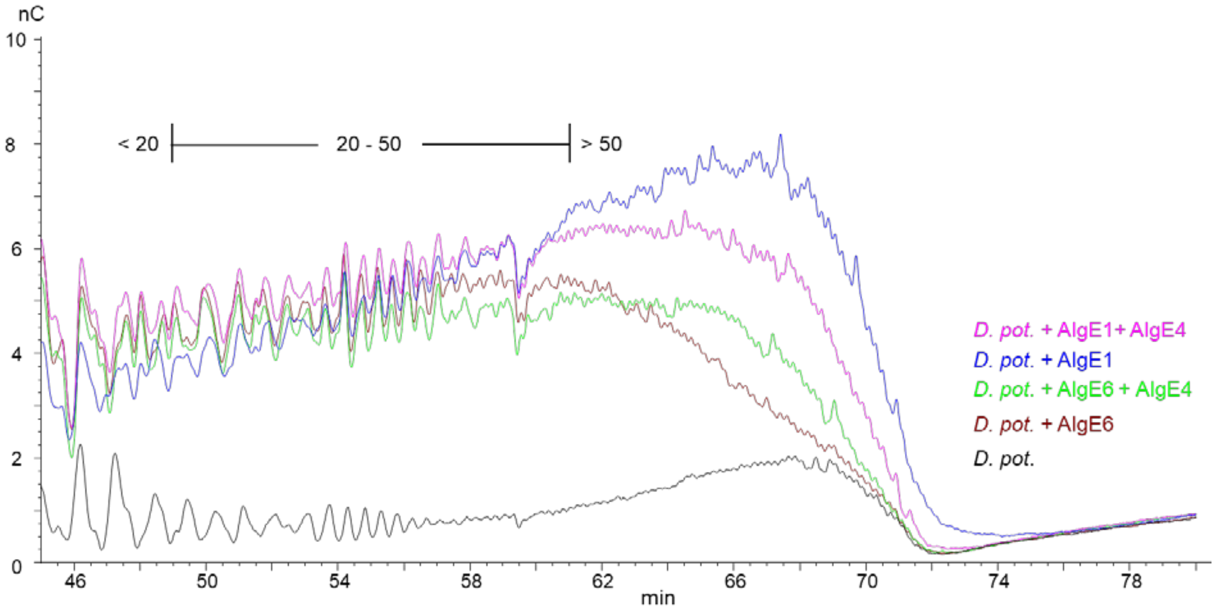

Figure S2. HPAEC-PAD chromatograms of alginate samples from *D. potatorum* before (black line) and after epimerisation with AlgE1 (blue line) and AlgE6 (brown line) alone and in combination with AlgE4 (pink and green lines). The epimerised alginate has been degraded with M-lyase prior to analysis thus leaving only the G-blocks. The scale bar above the chromatograms indicate the degree of polymerization (DP) eluting at the different time points i.e. DP below 20, DP 20-50 and DP > 50.

Table S1. Composition and sequential parameters of seaweed alginates before (without enzyme) and after epimerisation with AlgE1 and AlgE6 alone and in combination with AlgE4.

**Substrate**	**Epimerase**	F_G	F_M	F_{GG}	$F_{GM/MG}$	F_{MM}	$F_{GGM/MGG}$	F_{MGM}	F_{GGG}
L. hyperborea	without enzyme	0.49	0.51	0.33	0.16	0.35	0.04	0.12	0.29
	AlgE1	0.81	0.19	0.68	0.12	0.07	0.06	0.07	0.62
	AlgE1+AlgE4	0.81	0.19	0.69	0.13	0.06	0.06	0.08	0.62
	AlgE6	0.77	0.23	0.62	0.14	0.09	0.07	0.09	0.55
	AlgE6+AlgE4	0.78	0.22	0.65	0.14	0.08	0.06	0.08	0.58
L. nigrescens	without enzyme	0.42	0.58	0.24	0.18	0.39	0.06	0.16	0.18
	AlgE1	0.77	0.23	0.63	0.14	0.09	0.08	0.08	0.55
	AlgE1+AlgE4	0.78	0.22	0.63	0.15	0.08	0.08	0.08	0.55
	AlgE6	0.72	0.28	0.56	0.16	0.12	0.08	0.10	0.48
	AlgE6+AlgE4	0.75	0.25	0.60	0.16	0.09	0.08	0.09	0.52
A. nodosum	without enzyme	0.41	0.59	0.22	0.20	0.39	0.06	0.14	0.16
	AlgE1	0.74	0.26	0.57	0.17	0.10	0.09	0.08	0.48
	AlgE1+AlgE4	0.73	0.27	0.55	0.19	0.08	0.11	0.11	0.44
	AlgE6	0.69	0.31	0.51	0.18	0.13	0.10	0.09	0.42
	AlgE6+AlgE4	0.71	0.29	0.51	0.19	0.10	0.09	0.11	0.43
D. potatorum	without enzyme	0.32	0.68	0.20	0.12	0.56	0.05	0.07	0.16
	AlgE1	0.76	0.24	0.61	0.15	0.09	0.10	0.06	0.52
	AlgE1+AlgE4	0.77	0.23	0.61	0.16	0.07	0.10	0.08	0.51
	AlgE6	0.70	0.30	0.55	0.15	0.15	0.10	0.06	0.45
	AlgE6+AlgE4	0.74	0.26	0.58	0.16	0.10	0.10	0.07	0.48

Table S2. Molecular weight (analysed by SEC-MALLS) of alginates from brown algae used for epimerisation.

Sample	Mw (kDa)
L. hyperborea	250
L. nigrescens	240
A. nodosum	160
D. potatorum	230

Table S3 Agilent 8800 Series Triple Quadrupole ICP-MS System parameters.

**Parameter**	**Value**
RF Power	1550 W
Plasma Gas Flow	15 L/min
Auxiliary Gas Flow	0.9 L/min
Carrier Gas Flow	1.05 L/min
Option Gas Flow	0.0 L/min
Make Up Gas Flow	0.0 L/min
He Flow Rate	4.3 ml/min
O_2 Flow Rate	30%
Nebulizer Pump	0.1 rps
Sample depth	8.0 mm
Cell tuning modes	He/O2
S/C Temp	2°C
Scan Type	MS/MS
Replicate/peak pattern/sweeps	4/3/40
